# Alp/Enigma Family Proteins Cooperate in Z-Disc Formation and Myofibril Assembly

**DOI:** 10.1371/journal.pgen.1003342

**Published:** 2013-03-07

**Authors:** Anja Katzemich, Kuo An Liao, Stefan Czerniecki, Frieder Schöck

**Affiliations:** Department of Biology, McGill University, Montreal, Quebec, Canada; The Jackson Laboratory, United States of America

## Abstract

The *Drosophila* Alp/Enigma family protein Zasp52 localizes to myotendinous junctions and Z-discs. It is required for terminal muscle differentiation and muscle attachment. Its vertebrate ortholog ZASP/Cypher also localizes to Z-discs, interacts with α-actinin through its PDZ domain, and is involved in Z-disc maintenance. Human mutations in ZASP cause myopathies and cardiomyopathies. Here we show that *Drosophila* Zasp52 is one of the earliest markers of Z-disc assembly, and we use a Zasp52-GFP fusion to document myofibril assembly by live imaging. We demonstrate that Zasp52 is required for adult Z-disc stability and pupal myofibril assembly. In addition, we show that two closely related proteins, Zasp66 and the newly identified Zasp67, are also required for adult Z-disc stability and are participating with Zasp52 in Z-disc assembly resulting in more severe, synergistic myofibril defects in double mutants. Zasp52 and Zasp66 directly bind to α-actinin, and they can also form a ternary complex. Our results indicate that Alp/Enigma family members cooperate in Z-disc assembly and myofibril formation; and we propose, based on sequence analysis, a novel class of PDZ domain likely involved in α-actinin binding.

## Author Summary

Muscles are comprised of huge, multinucleated cells that feature a highly organized cytoskeletal architecture consisting of variable numbers of myofibrils, whose formation is not well understood. Each myofibril is an array of sarcomeres, the smallest contractile unit of muscles. The contractile system consists of actin filaments anchored at the Z-discs, which border the sarcomere, and myosin filaments anchored at the M-line in the middle of the sarcomere. In this study, we reveal the role of the Alp/Enigma family proteins Zasp52, Zasp66, and Zasp67 that are required for both the initial assembly and the stability of myofibrils. We also gain new insights into myofibril assembly by following it via live imaging. We can show that Zasp52 and Zasp66 cooperate in Z-disc assembly by binding directly to α-actinin, by interacting genetically, and by forming a ternary complex with α-actinin. As a result, the combined defects of removing both Zasp52 and Zasp66 or Zasp52 and another family member, Zasp67, are much more severe than would be expected from the additive defects of the single mutants. Thus, our results suggest that multiple Alp/Enigma family proteins are required to form the critical complex to initiate Z-disc and myofibril assembly.

## Introduction

Vertebrate muscles consist of three major types, skeletal, cardiac, and smooth muscles, which correspond in *Drosophila* to body wall, heart, and visceral muscle. Common to all is an actomyosin contractile system with thin filaments anchored at Z-discs. A crucial component of Z-discs is α-actinin, which anchors actin filaments at the Z-disc. In addition, proteins of the Alp/Enigma family function in maintenance of Z-discs [1] and have also been proposed to play an important role in myofibril assembly [2], [3]. In vertebrates, the Alp/Enigma family comprises ZASP/Cypher/LDB3/PDLIM6, ENH/PDLIM5, ENIGMA/PDLIM7, PDLIM1/CLP36, PDLIM2/Mystique, ALP/PDLIM3, and PDLIM4/RIL [4]. All vertebrate family members have one N-terminal PDZ domain, and one or three C-terminal LIM domains. In *Drosophila*, Zasp52 is a member of the Alp/Enigma family with a PDZ domain, Zasp-like motif and four LIM domains; another potential member, Zasp66, lacks the LIM domains, but features a similar PDZ domain and Zasp-like motif, and also localizes to Z-discs [2], [5]. Zasp52 was identified in an RNAi screen for spreading defects of S2R+ tissue culture cells [2], [6]. We could show that Zasp52 is a focal adhesion component and is required for cell spreading downstream of integrins. It also co-localizes with integrins at myotendinous junctions and is required for muscle attachment. Finally, it co-localizes with α-actinin to Z-discs and plays a role in embryonic Z-disc assembly [2]. Other groups proposed a role mainly in Z-disc maintenance [7], [8]. More recently we documented that Zasp52 occurs as at least 13 different splice isoforms and localizes to Z-discs in all muscle types in *Drosophila* [9]. Mutations of Zasp52 orthologs in vertebrates cause similar defects, ranging from improper formation of somites and heart in zebrafish to fragmented Z-discs in skeletal and cardiac muscles in mice [10], [11]. The single *C. elegans* ortholog ALP-1 displays defects in actin filament organization, but motility defects are much milder than in vertebrates or *Drosophila* [12], [13], [14]. Mutations in the human ortholog ZASP result in phenotypes of variable severity from congenital myopathy with fetal lethality to late-onset cardiomyopathy [1].

In this study, we show by live imaging of embryos that GFP-Zasp52 first assembles into repetitively spaced clusters, putative Z bodies, at the cortex of myotubes, which then coalesce to form Z-discs. We also show by antibody stainings that Zasp52 is among the first repetitively spaced Z-disc markers in indirect flight muscle (IFM) development, indicating that Zasp52 plays a general role in Z-disc assembly. We demonstrate a role for Zasp52, Zasp66, and the newly identified Zasp67 in IFM assembly, and show that Zasp52 acts together with Zasp66, both of which bind directly to α-actinin. Finally from sequence analyses we propose the name Zasp PDZ domain for PDZ domains with a putative α-actinin binding motif, which can be found in all vertebrate Alp/Enigma family members, as well as in Zasp66 and Zasp67 in *Drosophila*, and myopodin and CHAP in vertebrates.

## Results

### Live imaging of GFP-Zasp52 documents Z-disc assembly

**Figure 1:**
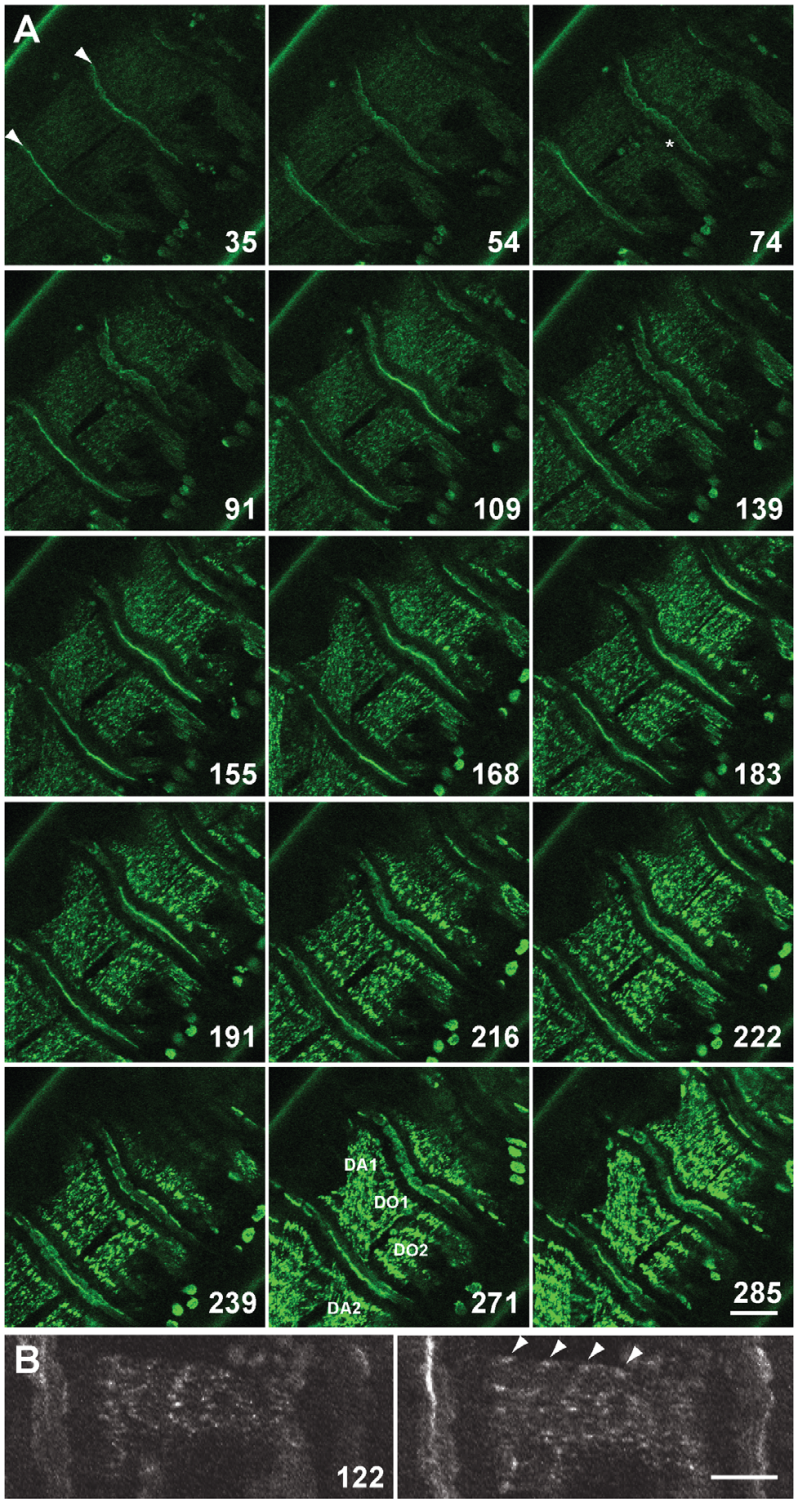
Live imaging of G00189 (GFP-Zasp52) during myofibril assembly of embryonic body wall muscles. (A) Z-disc assembly imaged over 300 min in a stage 16/17 embryo. Selected images up to 285 min are shown, for all images see Video S1. Time points shown are in minutes. Myotendinous junctions are marked with arrowheads at 35 min. Muscles DO1 and DO2 are imaged, although sometimes DA1 and DA2 are also visible due to muscle contractility (muscles are labeled at 271 min). GFP-Zasp52 gradually accumulates in clusters that steadily increase in size (compare e.g., 109, 168, and 216 min). GFP-Zasp52 is cleared first in areas next to myotendinous junctions (asterisk at 74 min). GFP-Zasp52 clusters sort out gradually into future Z-discs and eventually, GFP-Zasp52 clusters coalesce to form the final Z-disc (compare, e.g., 183, 222, and 285 min). Dorsal is in the upper left corner. Scale bar, 20 µm. (B) Enlarged view of DO2 muscle at 122 min. Left panel, surface view; right panel, a z-section 1 µm deeper. GFP-Zasp52 accumulations can be seen close to the sarcolemma in the myotube sagittal section (marked by arrowheads). Dorsal is up. Scale bar, 10 µm.

Zasp52 depletion causes partial embryonic lethality and defects in embryonic myofibril assembly, in particular Z-discs are not properly aligned and do not properly recruit α-actinin [2]. This suggests that Zasp52 could be an early marker suitable to follow myofibril assembly in real time. We also previously confirmed that line G00189 is a GFP-Zasp52 fusion that faithfully represents endogenous protein localization and is fully viable and functional [2]. We therefore used GFP-Zasp52 for live imaging to document Z-disc, and by extension, myofibril assembly (Figure 1A and Video S1). We focused on the period between late stage 16 embryos, when Zasp52 localizes only to myotendinous junctions and is evenly distributed within myotubes, and late stage 17 embryos, when Zasp52 localizes distinctly to both myotendinous junctions and Z-discs [2]. Our observations show: 1) GFP-Zasp52 gradually accumulates in clusters that steadily increase in size. 2) GFP-Zasp52 is first cleared from the area next to the myotendinous junctions. 3) GFP-Zasp52 clusters sort out gradually into future Z-discs. 4) GFP-Zasp52 clusters can first be observed close to the sarcolemma (Figure 1B). 5) Eventually, GFP-Zasp52 clusters coalesce to form the final Z-disc. 6) Sorting of GFP-Zasp52 clusters into future Z-discs correlates with a gradual increase in contractility, with initial contractility observed concomitant with GFP-Zasp52 clusters, after about 55 min in Video S1. These observations are consistent with our proposed model of myofibril assembly [3]. They demonstrate that GFP-Zasp52 is an early marker for myofibril assembly and a suitable tool for live imaging studies of myofibril assembly.

### Zasp52 Is Repetitively Arranged in Early Developing IFM

**Figure 2:**
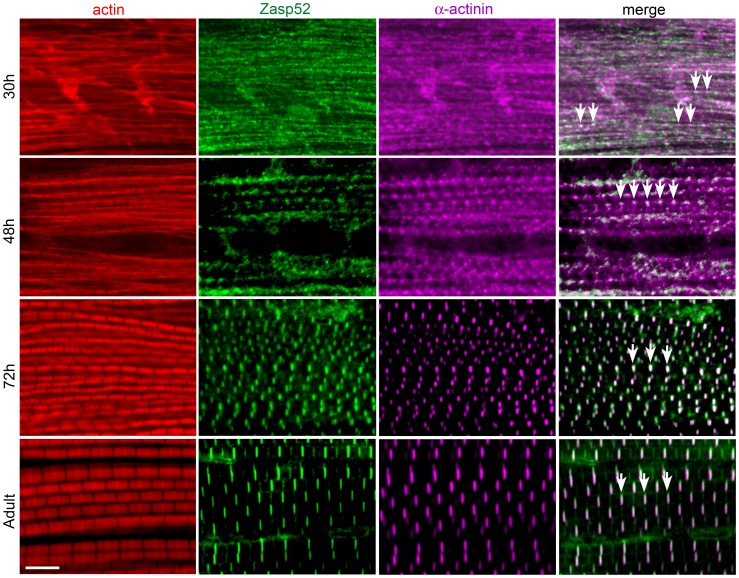
Zasp52 is an early Z-disc marker in IFM. Antibody staining of IFM at different stages of pupal development at 25°C, stained with phalloidin (red), anti-Zasp52 (green), and anti-α-actinin (magenta) antibody. At 30 h APF, Zasp52 and α-actinin have a punctate distribution along the forming myofibrils. Arrows indicate co-localization. At 48 h APF, Zasp52 and α-actinin appear as broad dots at the forming Z-disc. At 72 h APF, Zasp52 and α-actinin label in evenly spaced stripes at the Z-disc. In the adult, myofibrils have grown in width and Zasp52 and α-actinin are labeled in striations. Scale bar, 5 µm.

As Zasp52 localizes to Z-discs in all muscle types [9], we asked whether Zasp52 has a general role in Z-disc assembly. We therefore determined Zasp52 localization during IFM development. IFM development is distinct from that of embryonic body wall muscle in several respects. First, development takes much longer, approximately 96 h at 25°C from puparium formation to the emerging fly, and second, sarcomeres grow over time, from about 1.7 µm to a final length of 3.3 µm [15]. Due to the extended period of IFM development, we were able to perform antibody stainings at different stages of development. As during embryonic myogenesis, Zasp52 can be detected in a repetitive pattern at very early stages of pupal muscle development (Figure 2). At 30 h after puparium formation (APF), Zasp52 has a punctate distribution along the forming myofibrils and co-localizes with α-actinin. At this stage actin staining shows undifferentiated strands with no H-zones visible. At 48 h APF, Zasp52 and α-actinin appear as broad dots at the Z-discs. Myofibrils are narrow and actin labelling now shows a regular periodicity with evenly spaced H-zones. At 72 h APF, sarcomeres have grown in length, with Zasp52 and α-actinin appearing as elongated bands in the growing Z-disc. In the adult, Zasp52 and α-actinin are labeled in clear striations.

### Depletion of Zasp52 Long Isoforms Causes IFM Myofibril Defects

**Figure 3:**
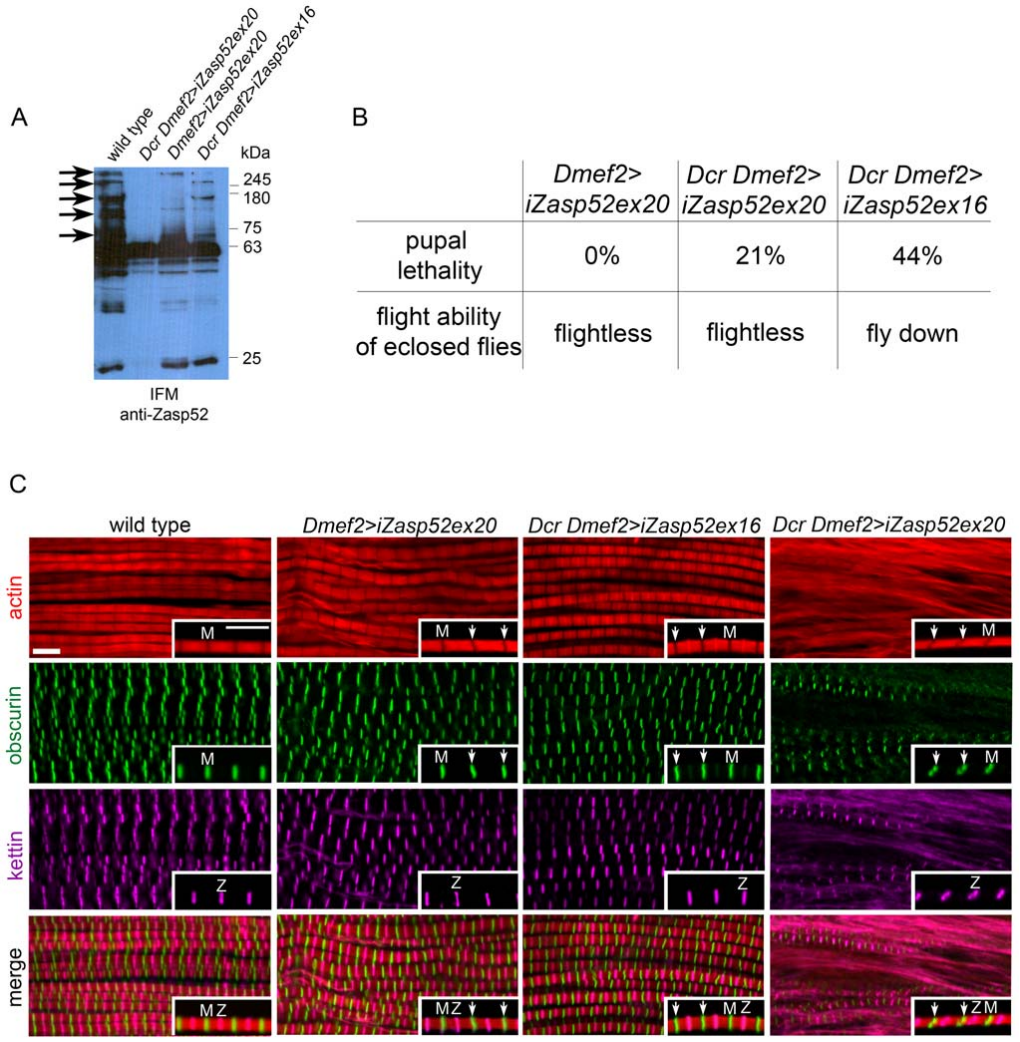
Depleting long isoforms of Zasp52 affects IFM structure. (A) Immunoblot of isolated IFMs from wild type and Zasp52 knockdown flies incubated with anti-Zasp52 antibody. Long Zasp isoforms are reduced in Dmef2>iZasp52ex20 flies and are absent in Dcr Dmef2>iZasp52ex20 flies. In Dcr Dmef2>iZasp52ex16 flies, long isoforms are reduced to a smaller degree compared to the other mutant genotypes. Arrows indicate several prominent depleted isoforms. (B) Pupal lethality and flight defects of wild type and iZasp52 knockdown flies. Pupal lethality is higher in Dcr Dmef2>iZasp52ex16 flies than in Dmef2>iZasp52ex20 and Dcr Dmef2>iZasp52ex20 flies. Dmef2>iZasp52ex20 flies are flightless, whereas Dcr Dmef2>iZasp52ex16 flies are flight impaired. (C) Adult IFM myofibrils of wild type and iZasp52 knockdown flies stained with phalloidin (red), anti-obscurin (green), and anti-kettin (magenta) antibody. Inserts show single myofibrils at higher magnification. Obscurin serves as an M-line and kettin as a Z-disc marker. In the wild type, H-zones are visible as straight dark lines. Obscurin and kettin label as straight bands in an alternating pattern at the Z-disc and M-line. In Dmef2>iZasp52ex20 flies, H-zones are occasionally bent and obscurin labels in wavy stripes (arrowheads). Frayed myofibrils are visible. Dmef2>iZasp52ex16 myofibrils also show distorted H-zones and wavy obscurin labeling. In Dcr Dmef2>iZasp52ex20 IFM, myofibrils are frequently frayed, H-zones are severely distorted, and obscurin and kettin labeling is abnormal. Scale Bar, 5 µm.

A strong hypomorphic mutation in *Zasp52* deleting most splice variants causes late embryonic to larval lethality [2]. We therefore decided to only deplete Zasp52 long isoforms with an RNAi transgene targeting the last exon of *Zasp52* (KK101276, called UAS-iZasp52ex20 in this study), which allows us to study the function of Zasp52 in IFM. We used the pan-muscle driver Dmef2-Gal4 to knock down Zasp52 in all muscles. We verified the knockdown efficiency by immunoblotting (Figure 3A). Long isoforms are almost absent in immunoblots from isolated IFM, and are completely gone with the addition of UAS-Dicer (Dcr), which enhances the generation of siRNAs. iZasp52ex20 mutant flies lacking long isoforms encoding LIM domains 2–4 are completely flightless (Figure 3B). To rule out off-target effects, we generated a second RNAi transgene against exon 16 with an shRNA transgene we call UAS-iZasp52ex16 (exon numbering according to [9]). Only by using Dcr, we were able to obtain a phenotype with this transgene. Dcr iZasp52ex16 mutants knock down long isoforms to a similar, but smaller degree as judged by immunoblotting (Figure 3A), and consistent with this, their flight ability is less impaired (Figure 3B).

We next analyzed adult IFM of iZasp52ex20 and Dcr iZasp52ex16 mutants by antibody staining and confocal microscopy (Figure 3C). In wild type myofibrils, obscurin and kettin, a titin isoform, label in straight bands at M-lines and Z-discs, respectively. H-zones are always straight and evenly spaced. In iZasp52ex20 and Dcr iZasp52ex16 knockdown flies, kettin labeling at the Z-disc appears normal, whereas obscurin in the M-line is occasionally wavy. Associated bent H-zones are frequently observed, indicating irregular thin filament lengths. In some areas of the IFM in iZasp52ex20 knockdown flies, unstable and frayed myofibrils are seen. This becomes more apparent when the phenotype is enhanced with Dcr showing myofibrils with distorted Z-discs and M-lines throughout the sample (Figure 3C). Overall, the phenotypes of both *Zasp52* RNAi transgenes are similar, consistent with them having no off-target effects. More importantly, the phenotypes indicate that Zasp52 is required for proper adult myofibril IFM structure, a muscle very different in structure and function from embryonic body wall muscles.

### Zasp52 Is Indispensable for Pupal Myofibril Development

**Figure 4:**
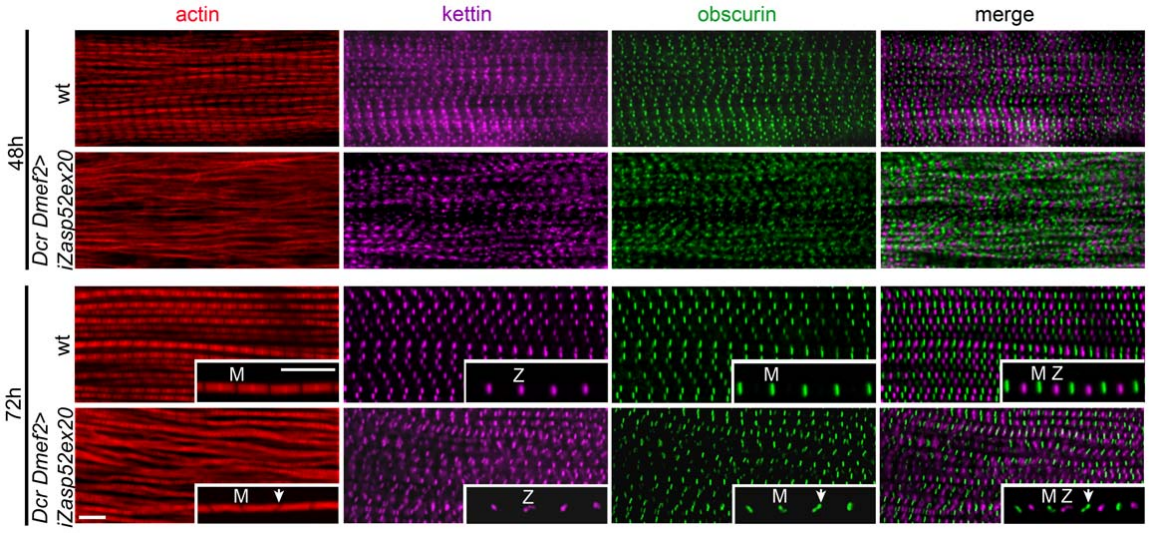
Zasp52 long isoforms are required for pupal myofibril development. Wild type and Dcr Dmef2>iZasp52ex20 IFM myofibrils stained with phalloidin (red), anti-obscurin (green) and anti-kettin (magenta) antibody at 48 and 72 h APF. Compared to wild type, myofibrillar defects are visible in Dcr Dmef2>iZasp52ex20 IFM at 48 h APF. Obscurin labeling in the M-line and kettin labeling in the Z-disc is less ordered compared to the wild type. Myofibrils are thinner and not well structured. At 72 h APF, some myofibrils are frayed and Z-discs and M-lines are deformed (arrowheads). Inserts show single myofibrils at higher magnification. Pupae were staged at 29°C. Scale Bar, 5 µm.

We wondered if Zasp52 defects arise already during development, or are maintenance defects due to muscle contractility of adult IFM. To address this question, we stained IFM muscles of wild type and Dcr iZasp52ex20 knockdown flies at different stages of pupal development (Figure 4). Disruptions of myofibrils of Dcr iZasp52ex20 knockdown flies become apparent at 48 h APF. At this stage myofibrils are thinner than in the wild type, without clearly defined H-zones. Kettin and obscurin label in fuzzy dots at Z-discs and M-lines, respectively. Kettin and obscurin appear less ordered than in the wild type, indicating that sarcomeres are not properly assembled. At 72 h APF, some of the myofibrils are frayed and kettin and obscurin labeling is in wavy stripes as seen in the adult fly. Overall these observations indicate that a lack of Zasp52 affects myofibril assembly during pupal development.

### A PDZ Domain Motif Putatively Involved in α-Actinin Binding

**Figure 5:**
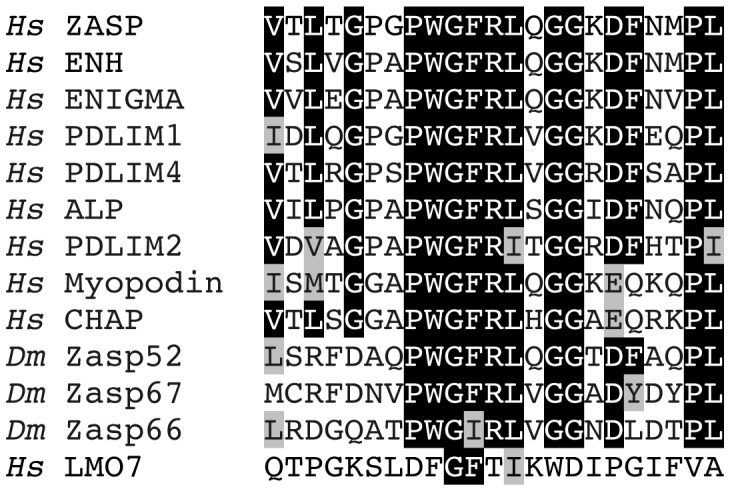
The Zasp PDZ domain. Alignment of 23 amino-terminal amino acids in the PDZ domain starting with V5 for ZASP. Mutagenesis demonstrated the importance of ZASP/Cypher residues G14 and F15 in binding α-actinin 2 [11]. A structural study predicted ZASP residues W13, G14, F15, R16, and L17 to form direct non-covalent interactions with the carboxy-terminal EF hands 3 and 4 of α-actinin 2 [16]. All human and *Drosophila* family members are shown and additionally LMO7, the closest relative that lacks this motif. Identical amino acids are highlighted in black, similar amino acids are highlighted in grey. *Hs*, *Homo sapiens*; *Dm*, *Drosophila melanogaster*.

We wondered if some functions of Zasp52 are masked by redundancy, and therefore performed a detailed database search with the Zasp PDZ domain among *Drosophila* and human proteins. We uncovered two *Drosophila* proteins, Zasp66 and CG14168, which we name Zasp67 owing to its cytogenetic location and similarity to Zasp66. Zasp66 and Zasp67 have a similar PDZ domain followed by the Zasp-like motif, and can therefore be classified as novel Alp/Enigma family members (Figure S4B). We also found two human proteins, CHAP and myopodin, which have a PDZ domain highly similar to Alp/Enigma family proteins with the putative amino acids required for α-actinin binding that are absent in the next-closest PDZ domain protein LMO7 (Figure 5 and Figure S1) [11], [16]. As CHAP and myopodin lack both LIM domains and the Zasp-like motif, we do not classify them as new members of the Alp/Enigma family. The PDZ domain-ligand interaction network was recently determined in humans [17]. Their algorithm predicts α-actinin as a likely ligand for all PDZ domains of this group, but not for LMO7 (Figure S1). Multiple sequence alignment and phylogenetic tree analysis shows that Zasp66 is the most distantly related member of this group of PDZ domains (Figure S2 and S3). We therefore decided to also investigate Zasp66, to obtain a better idea of functions potentially applying to all PDZ domains in this group. We also initiated characterization of Zasp67 to gather additional evidence on conserved functions of this protein family.

### Zasp66 Localization and Phenotype

Zasp66 was first identified with a GFP trap generating an endogenous GFP fusion protein demonstrating that Zasp66 localizes to Z-disks [5]. *Zasp66* is an alternatively spliced gene on chromosome arm 3L with at least 13 annotated transcripts (FlyBase; see Figure S4A for three representative transcripts). Zasp66 and Zasp52 both have very similar expression profiles with peak expression during embryonic and pupal myofibril assembly (FlyBase). They share an N-terminal PDZ domain and a Zasp-like motif that are highly similar to each other (Figure S4B). Four *Zasp66* transcripts encode both the PDZ domain and the Zasp-like motif, three transcripts encode a truncated PDZ domain and the Zasp-like motif, and the remaining six transcripts encode only the Zasp-like motif (Figure S4A).

**Figure 6:**
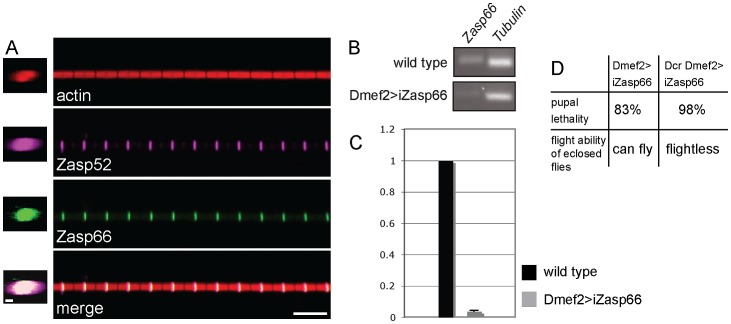
Zasp66 localization and iZasp66 phenotype. (A) Zasp52 and Zasp66 precisely co-localize at the Z-disc. Co-immunostaining of a Zasp66-GFP IFM myofibril with phalloidin and anti-Zasp52 antibody. Zasp66-GFP was detected by GFP fluorescence. Z-stacks on the left show Zasp52 and Zasp66 across the diameter of the myofibril. Scale bar left panels, 0.5 µm. Scale bar right panels, 5 µm. (B) RT-PCR of *Zasp66* and *Tubulin* from wild type and *Zasp66* RNAi knockdown flies at 29°C. (C) qPCR of *Zasp66*, *Tubulin*, and *rp49* from wild type and *Zasp66* RNAi knockdown flies at 29°C. Numbers on the y axis refer to averaged ratios of *Zasp66* mRNA to *Tubulin* and *rp49* mRNAs (normalized to 1 for wild type). (D) Phenotype of RNAi-mediated knockdown of *Zasp66* with or without Dcr scored at 29°C.

It is known that Z-disc or M-line proteins can distribute differentially throughout the diameter of their respective bands. For example, the vertebrate M-line proteins obscurin and Obsl1 localize in a non-overlapping pattern at the M-line, where obscurin is at the periphery of the myofibril, and Obsl1 in the core [18], [19]. We therefore co-immunostained GFP-Zasp66 with anti-Zasp52 antibody to determine if there are subtle differences in localization. Both co-localize indistinguishably throughout the entire diameter of the Z-disc (Figure 6A).

The genetic tools to analyze *Zasp66* are limited, because it is localized in a haploinsufficient region, and therefore no deficiencies are available. However, there is one hairpin RNA transgene (KK112973, called UAS-iZasp66 in this study) that targets a 200 bp *Zasp66* exon common to all 13 transcripts annotated by FlyBase (see Figure S4A). This construct has zero predicted off targets and should therefore knock down all known *Zasp66* transcripts with high specificity. We verified the knockdown of *Zasp66* with RT-PCR and qPCR demonstrating efficient knockdown (Figure 6B, 6C). Depletion of Zasp66 with Dmef2-Gal4 at 29°C results in high pupal lethality. With the inclusion of Dcr to enhance the phenotype, we observed almost complete pupal lethality and flightless adults (Figure 6D).

### Zasp66 Depletion Causes Developmental Defects Similar to Zasp52 Depletion

**Figure 7:**
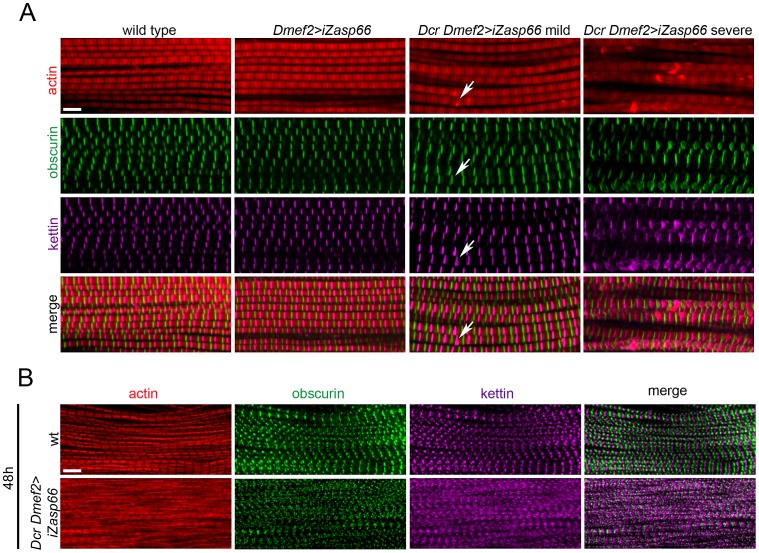
Zasp66 depletion affects adult IFM structure and pupal myofibril development. (A) Adult IFM myofibrils of wild type and *Zasp66* knockdown flies stained with phalloidin (red), anti-obscurin (green), and anti-kettin (magenta) antibody. Dmef2>iZasp66 myofibrils show no defects with perfectly arranged sarcomeres. The majority of Dmef2>iZasp66 flies enhanced with Dcr show mild myofibrillar abnormalities. Occasionally distorted M-lines and Z-discs are present (arrows). In more severe cases the integrity of sarcomeres is lost, and M-lines and Z-discs are severely disrupted. (B) IFM of pupae 48 h APF of wild type and Dcr Dmef2>iZasp66 flies stained with phalloidin (red), anti-obscurin (green) and anti-kettin (magenta) antibody. Myofibrils of Dcr Dmef2>iZasp66 IFM are less structured, similar to what was observed in Dcr Dmef2>iZasp52ex20 flies (Figure 4). Compared to wild type, obscurin and kettin appear less ordered. Scale bars, 5 µm.

We first investigated the IFM of rare adult escaper flies by confocal microscopy. They look largely normal with an occasional thickening and bending of Z-discs (Figure 7A, arrows). Rarely, we observed more severe disruptions in Z-disc structure (left panels in Figure 7A). Because 98% of Dcr iZasp66 knockdown flies die as pupae, we also investigated if developing IFM exhibit defects. We only analyzed the 48 h APF time point, because Dcr iZasp66 pupae at 72 h are largely dead or dying. Pupal IFM stained with phalloidin, anti-obscurin, and anti-kettin antibody reveal thinner and frayed myofibrils with irregular Z-discs (Figure 7B). Altogether, we examined 16 IFMs from individual 48 h knockdown pupae, all of which had a similar phenotype. These data indicate that Zasp66, like Zasp52, contributes to Z-disc assembly during development.

### Zasp66 and Zasp52 Both Contribute to Adult Z-Disc Stability

**Figure 8:**
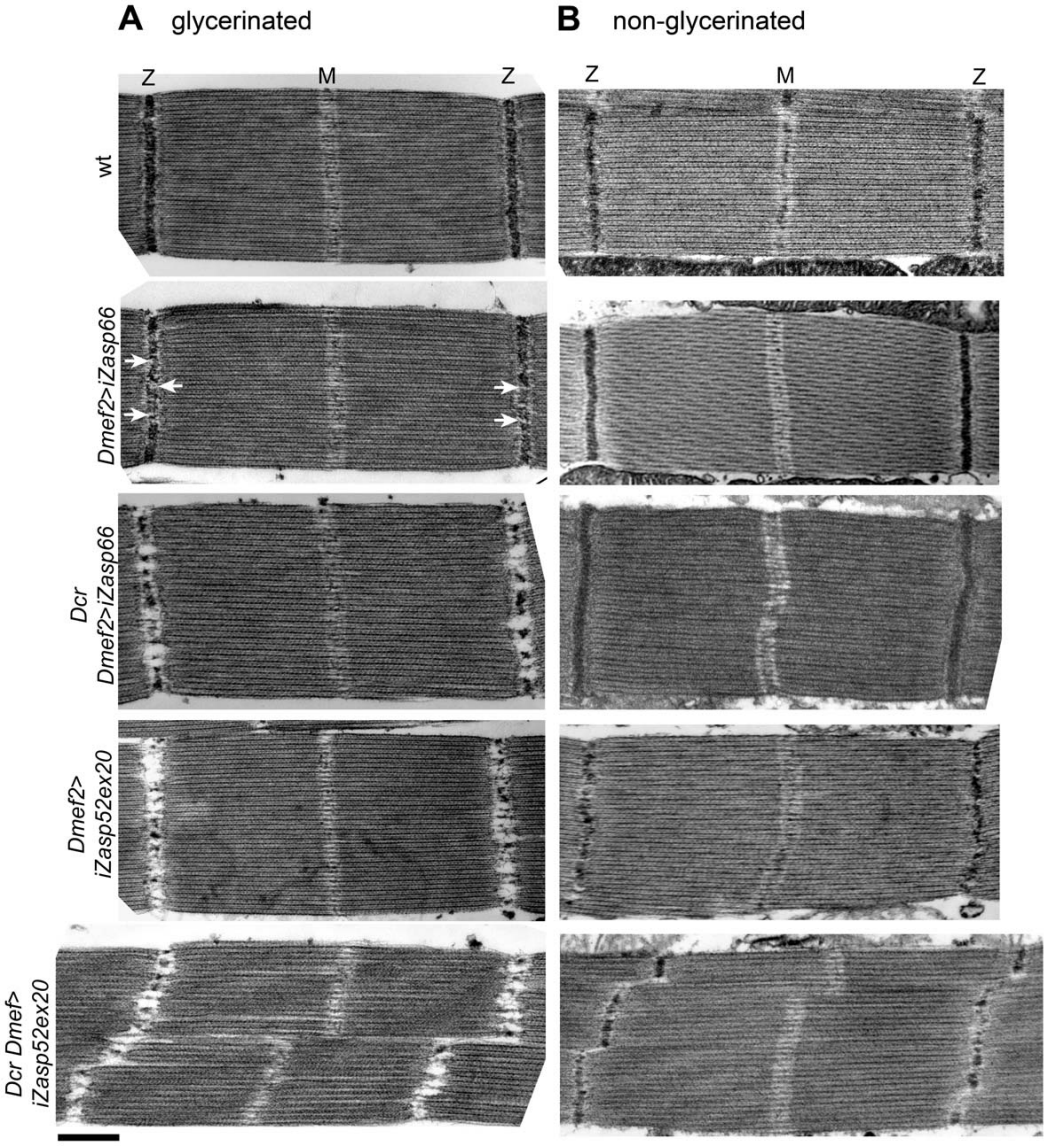
Zasp66 and Zasp52 stabilize Z-discs. Electron micrographs of wild type, Dmef2>iZasp52ex20, and Dmef2>iZasp66 IFM with and without Dcr. (A) Sarcomere structure of IFMs treated with glycerol and Triton-X 100. Dmef2>iZasp66 sarcomeres show mild defects in their Z-disc structure. Small pockets with missing Z-disc material are visible (arrows). Sarcomeres of Dcr Dmef2>iZasp66 flies have little Z-disc material left, similar to sarcomeres of Dmef2>iZasp52ex20 flies. Sarcomeres of Dcr Dmef2>iZasp52ex20 flies show severely distorted Z-discs and H-zones. (B) Sarcomere structure of IFMs without glycerol and Triton X-100 treatment. Dmef2>iZasp66 sarcomeres look like wild type. Sarcomeres of Dmef2>iZasp66 flies enhanced with Dcr occasionally show shifted H-zones and slightly bent Z-discs. Dmef2>iZasp52ex20 sarcomeres with or without Dcr lack Z-disc material, but show a milder phenotype than in (A). Scale bar, 500 nm.

We next asked if Zasp66 also contributes to Z-disc stability. Given that hatched flies without Dcr enhancement could fly, we did not expect strong defects, and therefore we compared sarcomeric organization of IFM by transmission electron microscopy (TEM). Zasp66 RNAi mutants exhibit mild defects in Z-disc structure (Figure 8A). Wild type Z-discs are always completely regular, whereas *Zasp66* mutant Z-discs often show small pockets, where Z-disc material is missing. These defects are weaker than defects caused by depletion of iZasp52ex20. When long isoforms of Zasp52 are depleted, there is very little Z-disc material left (Figure 8A). Apart from occasionally shifted H-zones, the rest of the sarcomere is unaffected, with correctly arranged thick and thin filaments. As seen by confocal microscopy, this phenotype is enhanced by the use of Dcr, with frequently distorted Z-discs and H-zones. In the case of Zasp66, the use of Dcr results in a phenotype similar in strength to the phenotype observed when iZasp52ex20 is depleted without Dcr. As in the majority of cases the overall integrity of sarcomeres was not lost, we asked if Z-discs are unstable and fall apart due to the conditions used for sample preparation for TEM. During sample preparation, IFMs were treated with glycerol and Triton X-100 in order to be able to place sarcomeres into rigor state. This allows detailed structural analysis of sarcomere components and normally does not interfere with sarcomere architecture [20]. We analyzed iZasp66 and iZasp52ex20 mutant IFM without the use of glycerol and Triton X-100. In this case the Z-discs of iZasp52ex20 knockdown flies appear more complete, but are much thinner with many irregularities. iZasp66 Z-discs look like wild type (Figure 8B). This demonstrates that both Zasp52 and Zasp66 are crucial for Z-disc stability.

### Zasp52 Forms a Ternary Complex with α-Actinin and Zasp66, and Genetically Interacts with Zasp66

**Figure 9:**
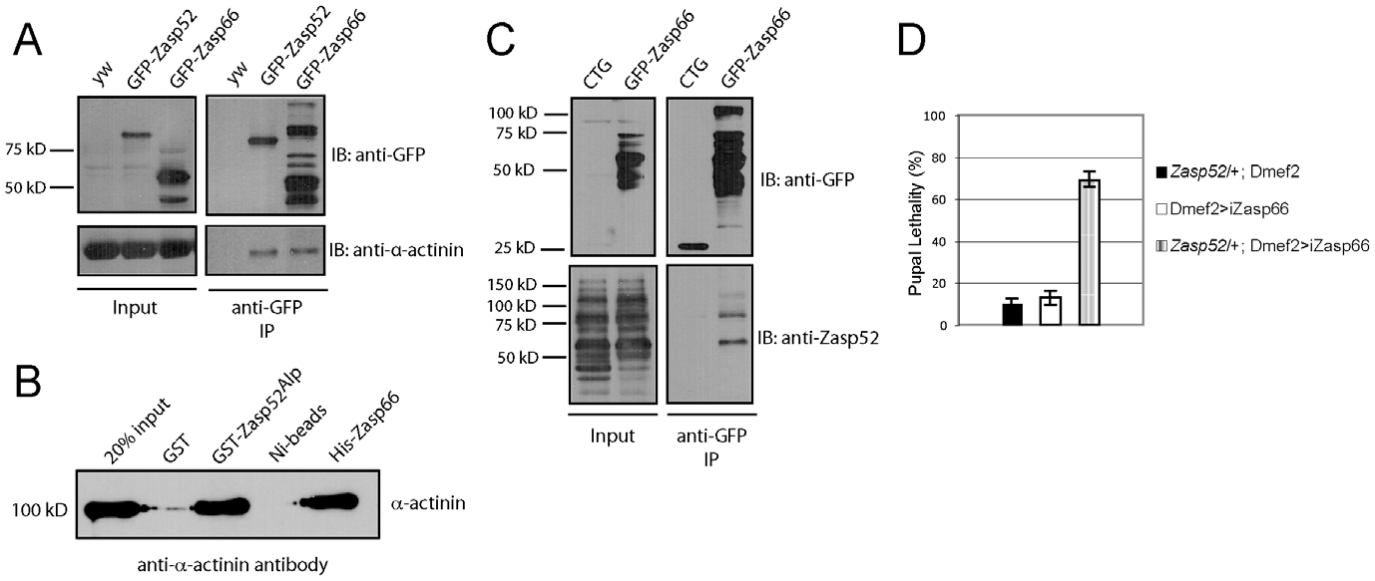
Zasp52 and Zasp66 both bind α-actinin and genetically interact. (A) Immunoprecipitation of GFP-Zasp52 and GFP-Zasp66 with anti-GFP beads pulls down α-actinin. (B) Direct interaction between GST-tagged Zasp52^Alp^ and α-actinin by GST pull-down and His-tagged Zasp66 and α-actinin by Ni-NTA pull-down. Pulled down α-actinin was detected by immunoblot analysis using anti-α-actinin antibody. (C) Immunoprecipitation of Zasp66-GFP pulls down Zasp52, while mesodermally expressed GFP alone (CTG: twi-Gal4 UAS-EGFP) does not. (D) Genetic interaction assay testing pupal lethality at 25°C. Removing one copy of *Zasp52* in addition to *Zasp66* knockdown causes strong synergistic lethality.

We finally wanted to know if and how Zasp52 and Zasp66 act together in Z-disc assembly. We showed previously that Zasp52 can co-immunoprecipitate α-actinin [2], and in vertebrate ZASP/Cypher, the PDZ domain is crucial for interaction with α-actinin [11], [21], [22]. Zasp52 and Zasp66 both carry an N-terminal PDZ domain. We therefore tested whether both Zasp52 and Zasp66 can bind α-actinin by pulling down endogenous GFP fusions to Zasp52 and Zasp66 with anti-GFP antibody-coupled beads. Both GFP-Zasp52 and GFP-Zasp66 robustly co-immunoprecipitate α-actinin, whereas extracts from *y w* control flies do not (Figure 9A). We next asked if this interaction is direct. We overexpressed and purified His-tagged Zasp66 and GST-tagged Zasp52 from bacteria, and tested direct interaction with rabbit skeletal muscle α-actinin. Both Zasp52 and Zasp66 directly interact with α-actinin (Figure 9B). We can also show that GFP-Zasp66 co-immunoprecipitates Zasp52, whereas control flies expressing GFP alone do not (Figure 9C). These data indicate that Zasp52, Zasp66 and α-actinin form a ternary complex.

Finally, we determined if *Zasp66* genetically interacts with *Zasp52*. To this end, we removed one copy of *Zasp52* in the background of Zasp66 depletion by RNAi and measured pupal lethality. While knocking down Zasp66 on its own or only removing *Zasp52* heterozygously shows mild pupal lethality at 25°C, removing *Zasp66* together with one copy of *Zasp52* substantially increases pupal lethality, demonstrating a genetic interaction between *Zasp52* and *Zasp66* (Figure 9D). These data indicate that Zasp52 and Zasp66 cooperate in Z-disc assembly and that both are direct binding partners for α-actinin.

### Zasp66 Zasp52 Double RNAi Knockdown Dies Earlier and Has a More Severe Phenotype

**Figure 10:**
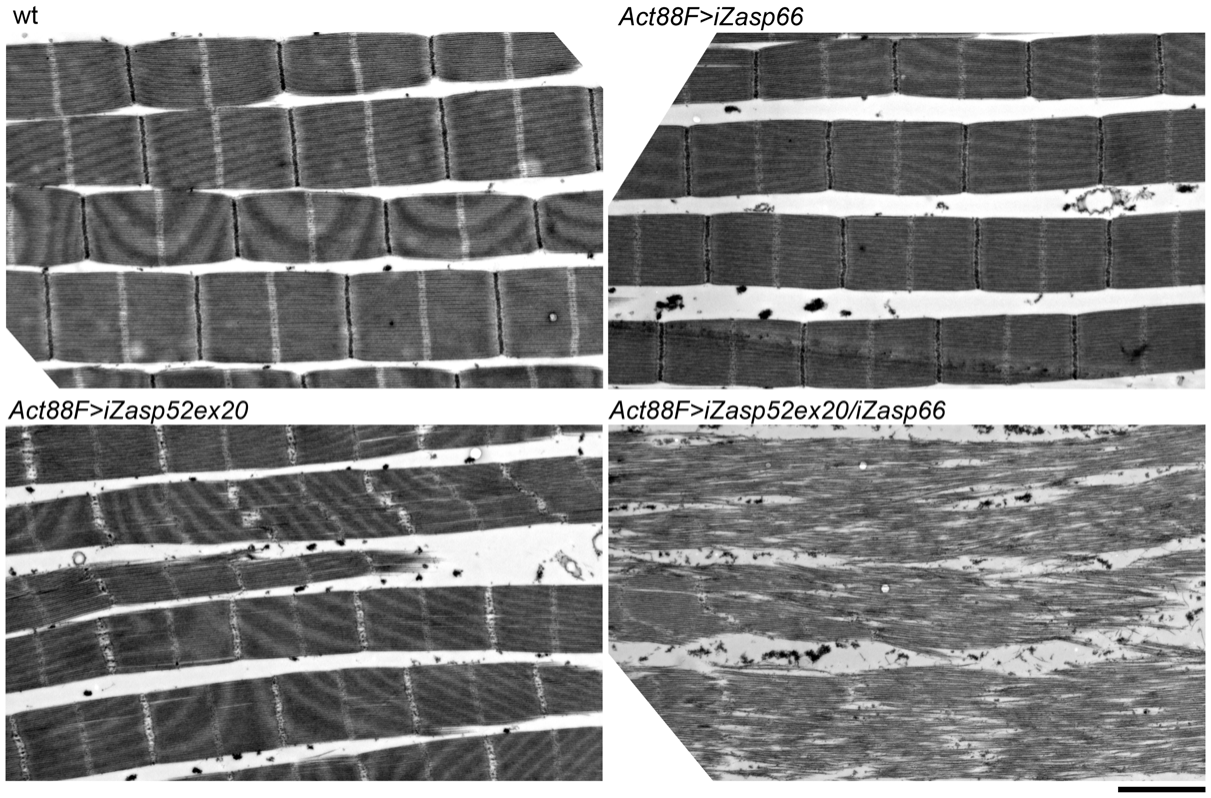
Zasp52 and Zasp66 cooperate to assemble myofibrils. Electron micrographs of IFM of wild type, Act88F>iZasp66, Act88F>iZasp52ex20, and Act88F>iZasp52ex20/iZasp66 double mutants. Global views are shown. The double mutant shows a severe disruption of sarcomere structure, at the level of the Z-disc as well as the filament system, where fraying and misaligned thick and thin filaments can be observed. Scale bar, 2 µm.

To address the issue of potential redundancy between Zasp52 and Zasp66 in Z-disc assembly, we investigated the double mutant phenotype. Using the pan-muscle driver Dmef2-Gal4, iZasp52ex20 and iZasp66 double mutants die at the earliest stage of pupal development precluding analysis of developing IFM. We therefore used the IFM-specific driver Act88F-Gal4 [23], in order to obtain adult double knockdown flies. As with Dmef2-Gal4, iZasp66 knockdown flies are able to fly and show no severe phenotype when analyzed by electron microscopy using glycerol and Triton X-100 extraction (Figure 10). Sarcomeres are properly arranged and Z-discs have small pockets with missing Z-disc material. In iZasp52ex20 single knockdown flies the phenotype was as observed when driven with Dmef2-Gal4. There is almost no Z-disc material left and H-zones and Z-discs are distorted occasionally. No frayed myofibrils can be seen (Figure 10). In iZasp52ex20 iZasp66 double knockdown flies, we observe a more severe phenotype than would be expected by additive effects of single knockdowns. Myofibrils are frequently frayed and unstable, with severely distorted Z-discs and H-zones (Figure 10). This synergistic defect indicates that Zasp52 and Zasp66 function partially redundantly during myofibril assembly in the IFM and cooperate in stabilizing Z-discs.

### IFM Phenotype of Zasp67 RNAi Knockdown Is Similar to Zasp52

*Zasp67* is exclusively expressed during pupal stages at a time when pupal myofibrils assemble (FlyBase), but we do not know if Zasp67 protein localizes to Z-discs similar to Zasp52 and Zasp66. We tested two available Zasp67 RNAi transgenes, GD8245 and KK111478, which both result in flight-impaired flies, when expressed in muscles with Dmef2-Gal4. We continued to work with KK111478, which we call iZasp67. We can show by RT-PCR and qPCR that *Zasp67* is efficiently knocked down (Figure S5B, S5C). We then characterized IFMs of iZasp67 mutants by electron microscopy. They exhibit a phenotype very similar to knocking down the long isoforms of Zasp52 (Figure S5A). We also analyzed *Zasp67 Zasp52* double mutants, which look similar to *Zasp66 Zasp52* double mutants, but even more severe (Figure S5A). We lastly checked α-actinin localization in various mutant combinations, which all still express one or several Zasp isoforms. We observe normal α-actinin localization in all mutant combinations (Figure S6, see Discussion). These results indicate that Alp/Enigma family members in *Drosophila* act partially redundantly in the same pathway, the assembly of Z-discs.

## Discussion

In this study we show that Zasp52 marks Z-disc assembly in two widely differing muscle types, adult IFM and embryonic body wall muscles. During embryonic body wall assembly, GFP-Zasp52 delineates steps previously outlined in myofibril assembly models [3], [24], [25]. In IFM assembly, Zasp52 localizes to developing Z-discs at the earliest stages and is required for development and stability of myofibrils. We also uncover two other, closely related proteins, Zasp66 and Zasp67, which function together with Zasp52 in myofibril assembly. We show that two of these, Zasp52 and Zasp66, bind directly to α-actinin, prompting us to propose by sequence analysis a new class of PDZ domains closely related to that of vertebrate ZASP/Cypher with the putative ability to bind α-actinin.

### Myofibril Assembly in Embryos

Our live imaging with GFP-Zasp52 is the first time-lapse recording of myofibril assembly in a whole animal. The only other live imaging in whole animals was done in zebrafish skeletal muscle for fluorescence recovery after photobleaching [26]. Other time-lapse recording studies used reassembly of myofibrils in tissue culture cells [27]. A further difference to previous studies is that GFP-Zasp52 is endogenous, fully functional and viable. Still, both our and previous studies agree on two points. First, Z-disc proteins initially form separate clusters close to the sarcolemma, presumably corresponding to the Z bodies described in electron microscopy studies. Second, these clusters coalesce and align into Z-discs. Our study additionally documents that GFP-Zasp52 clusters are initially evenly distributed and gradually sort out to the future Z-disc, while at the same time growing in size. We also show a clear correlation between Z-disc assembly and an increase in contractility. We notice one important difference: in avian heart and in zebrafish skeletal muscle as well as in IFM, myofibril assembly is approximately an order of magnitude slower than in the *Drosophila* embryo. Also, non-embryonic myofibril assembly involves an increase in sarcomere length, or a premyofibril stage, which employs non-muscle myosin [15], [28]. In embryonic myofibril assembly, there is no increase in sarcomere length, and initial spacing corresponds to the final sarcomere length. This is likely due to time constraints of the very fast development of *Drosophila* embryos. This timelapse study fits very well with a model we have proposed recently [3], and also with a computational modeling study indicating that actin clusters cross-linked at the barbed end (Z bodies) together with actin filament treadmilling is sufficient for establishment of sarcomere arrays [29]. In the IFM, a very different muscle type, Zasp52 also localizes to Z-disc precursors at the earliest stages of pupal IFM development, further strengthening the notion that myofibril assembly is highly conserved across muscle types. Live imaging of myofibril assembly in *Drosophila* embryos provides a suitable model system, because it occurs very quickly, and without the complications of sarcomere growth.

### Zasp52, Zasp66, and Zasp67 Contribute to Z-Disc Assembly

The adult *Zasp52* IFM phenotypes confirm and extend our previous observations on body wall muscles of embryos [2]. We used UAS-iZasp52ex20 to study *Zasp52* phenotypes in IFM. It will form a 573 nt hairpin targeting the last exon of *Zasp52* [30]. Even though no off-targets are predicted for this construct, we wanted to independently verify our phenotype with a different construct, and therefore generated an shRNA construct targeting only 19 nt within exon 16 (UAS-iZasp52ex16), also without predicted off-targets. As judged by immunoblotting (Figure 3A), both constructs target only the long isoforms of Zasp52, though Dcr iZasp52ex16 is slightly less efficient. They produce similar IFM defects, with the slightly weaker phenotype of Dcr iZasp52ex16 being consistent with its apparent reduced knockdown efficiency (Figure 3B, 3C). Surprisingly, Dcr iZasp52ex16 causes stronger pupal lethality (Figure 3B). This could be due to an off-target effect, a stronger reduction of a critical embryonic or larval isoform with iZasp52ex16, or the difference between knocking down all long isoforms versus exon 16-containing long isoforms. Muscle defects are very similar with both the pan-muscle driver Dmef2-Gal4 and the IFM-specific Act88F-Gal4 driver (Figure 8, Figure 10). This is consistent with inefficient knockdown of Zasp52 long isoforms during larval stages using Dmef2-Gal4 (A. K., unpublished observations).

We demonstrate that the stability of Z-discs is severely compromised upon depletion of Zasp52 or Zasp66, because significant amounts of Z-disc proteins can be lost simply by detergent extraction (Figure 8). The impaired stability likely gives rise to the misalignment of Z-discs and H-zones that we observe during IFM myofibrillogenesis (Figure 4). If IFM muscles contract during assembly or are under tension as we show for embryonic body wall muscles, then unstable Z-discs should lead to the misalignment of thin and thick filaments, resulting in wavy H-zones and M-lines. These developmental defects appear very early, consistent with our imaging data on embryonic myofibril assembly and our proposed role for Zasp52 as an organizer for Z body assembly [3]. We observe a similar developmental defect in Zasp66-depleted pupal IFM myofibrils (Figure 7B). The Z-disc defects in single knockdowns of Zasp52 long isoforms are also similar to α-actinin mutants [31], supporting the interdependence of Zasp PDZ domain proteins and α-actinin at the Z-disc. We still observe α-actinin at the Z-disc in various mutant combinations (Figure S6). We do not believe that this result is contradictory to our previous observation of reduced α-actinin recruitment to Z-discs in embryonic body wall muscles [2], because even in double mutants, there are still several Zasp proteins expressed. For example, the most severe double mutant (iZasp52ex20 iZasp67) still expresses the short Zasp52 isoforms and all Zasp66 isoforms. Moreover, the double mutant phenotypes are much more severe than the α-actinin null mutant phenotype [31], consistent with Zasp proteins being upstream of α-actinin in Z-disc assembly. Finally, the phenotypic features we see in flies are similar to human myopathies [32], supporting the use of fly muscles as a model system.

Importantly, the *Zasp52 Zasp66* double mutant phenotype is considerably more severe than would be expected from additive defects of single knockdowns (Figure 10), and the same is true for the *Zasp52 Zasp67* double mutant (Figure S5), indicating a synergistic mechanism, where Zasp52, Zasp66, and Zasp67 cooperate in Z-disc assembly. A possible mechanism is the formation of a multiprotein complex consisting of α-actinin, Zasp52, Zasp66, and Zasp67 at the forming Z-disc, which helps in assembly and stabilization of the Z-disc. The genetic interaction of Zasp52 and Zasp66 and the direct binding of Zasp52 and Zasp66 to α-actinin support this model (Figure 9). Our results suggest that several Zasp-like proteins are required together with α-actinin to form a critical complex for Z body and Z-disc assembly. Such multiprotein complexes have already been reported for ENH, Cypher, calsarcin and myotilin, and have been inferred from RNAi studies for Zasp52, non-muscle myosin and α-actinin [8], [33].

### The Zasp PDZ Domain

The Alp/Enigma family comprises ZASP, ENH, ENIGMA, PDLIM1, PDLIM2, ALP, and PDLIM4, and characteristically contains an amino-terminal PDZ domain, a Zasp-like motif, and carboxy-terminal LIM domains [4]. The *Drosophila* ortholog with the same domain organization is Zasp52, also called Zasp [2], [5]. We propose to include *Drosophila* Zasp66 and Zasp67 as novel family members, because they share a similar amino-terminal PDZ domain followed by the characteristic Zasp-like motif (Figure S4B). Zasp66 and Zasp67 do not encode LIM domains, however, both Zasp52 and other Alp/Enigma family members encode protein isoforms without LIM domains [4], [9], [34], indicating the existence of functional Alp/Enigma proteins without LIM domains. We uncovered two additional proteins, myopodin and CHAP, with a highly related PDZ domain (Figure 5). These proteins lack both LIM domains and the Zasp-like motif, therefore they are likely not Alp/Enigma family members. We propose the name Zasp PDZ domain for PDZ domains with an amino-terminal PWGFRLxGG motif, which is likely required for α-actinin binding [11], [16]. We chose the name Zasp PDZ domain for two reasons: 1) the first PDZ domain that was crystallized and functionally analyzed, is from ZASP/Cypher [16], [21], [22]. 2) ZASP was also the first gene for which mutations in humans causing myopathies were identified [1], [32], [35], [36].

Six of the proteins with a Zasp PDZ domain, ZASP, ENH, PDLIM1, PDLIM2, Alp, and PDLIM4 bind α-actinin via their PDZ domain [21], [22], [37]–[41]. In addition, ZASP, Enigma, ENH, PDLIM1, and Alp localize to Z-discs [21], [22], [37], [39], [42]–[44], while PDLIM2 and PDLIM4 localize to actin stress fibers in non-muscle cells [38], [40]. CHAP plays an important role in myofibril assembly and co-localizes with α-actinin, but whether the PDZ domain is involved in α-actinin binding has not been clarified [45]. For myopodin, only an isoform that lacks the PDZ domain and functions in skeletal muscles has been analyzed [46], but heart muscle expresses a 95 kD isoform localizing to Z-discs that could correspond to a PDZ-containing isoform [47]. Given that the most diverging Alp/Enigma protein in this group, Zasp66 (see Figure 5 and Figure S3), as well as Zasp52, also interact directly with α-actinin (Figure 9B), it appears likely that all proteins containing a Zasp PDZ domain can do so. In contrast, the closest relative, LMO7, cannot bind α-actinin through its PDZ domain [48]. Finally, the Alp/Enigma family proteins ZASP, PDLIM1, and ALP contain an additional area partially overlapping with the Zasp-like motif, which interacts with the α-actinin rod domain, giving rise to the possibility that one Zasp molecule may bind two α-actinin dimers or one α-actinin dimer in antiparallel configuration [43], [49]–[51].

Recently, a comparative evolutionary study showed that only four ortholog groups localize to Z-discs in all bilaterian species: Zasp, α-actinin, titin, and MLP proteins, suggesting that these four protein groups could be sufficient for assembly and function of Z-discs [52]. Our results indicate that multiple members of Zasp PDZ domain proteins may be required to provide the critical mass for Z body assembly. Together with the well-documented role of ZASP mutations in human disease, our data indicate that these proteins occupy a central place in muscle assembly and function.

## Materials and Methods

### Fly Stocks and Genetics

The following fly stocks were used: G00189 (GFP-Zasp52), zcl0663 (GFP-Zasp66), Dmef2-Gal4, UAS-Dcr Dmef2-Gal4, *Gla*/CTG [CyO, P{GAL4-twi.G}2.2, P{UAS-2×EGFP}AH2.2] from the Bloomington *Drosophila* Stock Center, *Zasp52* [2], Act88F-Gal4 [23] (kindly provided by RM Cripps), UAS-iZasp66 (KK112973, transformant 102980), UAS-iZasp52ex20 (KK101276, transformant 106177), UAS-iZasp67 (GD8245, transformant 17414 and KK111478, transformant 103225) from the Vienna *Drosophila* RNAi Center, and UAS-iZasp52ex16 (this study). Act88F-Gal4; UAS-iZasp66 was generated by standard genetic crosses.

For the genetic interaction assay, UAS-iZasp66 was crossed to Dmef2-Gal4, and *Zasp52*/CTG; Dmef2-Gal4 was crossed to UAS-iZasp66 or *y w* and incubated at 25°C. After 14 days of incubation, pupal lethality was scored (ratio of non-green pupae to straight-winged adults).

### Live Imaging

Live imaging was performed as described [53]. Briefly, two-hour egg-lays were aged for 24 h at 18°C to obtain late stage 16 embryos. Embryos were dechorionated in 50% bleach for 2 min, rinsed, dried and mounted in halocarbon 27 oil on a gas-permeable membrane (Coy Lab Products, MI, USA). Micrographs were taken on a Zeiss LSM 510 Meta laser scanning confocal microscope at room temperature with a 40×1.3 Plan-NEOFLUAR oil immersion objective at 2× zoom. Every minute, 7 z-sections were captured at 512×512 resolution, 2× scan average, with each slice being separated by 1 µm (total scan time: 14 sec). After collection, sections were separated and exported as TIFF files using Volocity software (PerkinElmer, Ontario, Canada).

### Molecular Biology

For RT-PCR and qPCR, the average of two independent experiments of triplicate-PCR reactions is presented. Total RNA was isolated from 20 adult flies using Trizol, and reverse transcribed using SuperScript II Reverse Transriptase according to the manufacturer's instructions (Life Technologies, Ontario, Canada) and run on a T3000 Thermocycler for RT-PCR (Biometra, Montreal Biotech Inc., Quebec, Canada). Quantitative PCR reactions were performed with the iQ SYBR Green Supermix Kit on a C1000 Thermocycler (Bio-Rad, Ontario, Canada). Quantification was performed with the comparative threshold cycle method on Bio-Rad CFX Manager software. Both *rp49* and *β-Tubulin* were used as normalization controls in a single experiment. Primer pairs used: Zasp66-F TACCGTACAACTCCGCTGGT, Zasp66-R TCATGGTAGTCGTGTCCTGG, Zasp67-F CTTAATGGTGGGCAGCAAGTC, Zasp67-R GACAGTGAGGTGCCGAATTT, tubulin-F ACATCCCGCCCCGTGGTC, tubulin-R AGAAAGCCTTGCGCCTGAACATAG, rp49-F TACAGGCCCAAGATCGTGAAG, rp49-R GACGCACTCTGTTGTCGATACC. Zcl0663 (GFP-Zasp66) was verified by PCR with primer pair GFP-fwd CGACCACTACCAGCAGAACA and Zasp66-rev GATGCACCTACGCCACTTTT. For UAS-iZasp52ex16 we designed oligos generating a 21 nt siRNA targeting exon 16 with the DSIR algorithm (http://biodev.extra.cea.fr/DSIR/DSIR.html). It should deplete all long isoforms except one containing exon 17 (exon numbering according to [9]. Oligos ctagcagtCTGCACATTGCAGCTGTTGCAtagttatattcaagcataTGCAACAGCTGCAATGTGCAGgcg and aattcgcCTGCACATTGCAGCTGTTGCAtatgcttgaatataactaTGCAACAGCTGCAATGTGCAGactg were annealed and cloned into Valium20 [54]. A sequence-verified clone was injected into *vermillion attP2*(3L) flies (Genetic Services Inc., MA, USA).

### Immunoprecipitation and Binding Assays

50 adult fly thoraces were cut in half and were homogenized in lysis buffer (25 mM Tris-HCl pH 8, 150 mM NaCl, 1 mM EDTA, 0.5% TritonX-100, 5% glycerol, and complete EDTA-free Protease inhibitor cocktail; Roche, Quebec, Canada). Protein extracts were then incubated with prewashed GFP-Trap-M anti-GFP beads (ChromoTek, Germany) for 2 h at 4°C. After incubation the beads were washed three times with wash buffer (10 mM Tris-HCl pH 8, 150 mM NaCl, 0.1% TritonX-100 and complete EDTA-free Protease inhibitor cocktail), and bound proteins were eluted by boiling in 2× SDS sample buffer. Eluates were analyzed by SDS-PAGE and by immunoblotting. Antibodies were used at the following ratios: rat anti-α-actinin antibody at 1∶2000 (Babraham Institute, UK); rabbit anti-GFP antibody at 1∶400 (Clontech, CA, USA). The immunoreaction was visualized by ECL (GE Healthcare, Ontario, Canada).

Zasp66-RB was synthesized by GenScript (New Jersey, USA), and cloned into pRSETA (Life Technologies, Ontario, Canada); GST-Zasp52^Alp^ (amino acids 1–357 containing the PDZ domain, the Zasp-like motif and the LIM1 domain) was cloned from EST LP01550 into pGEX-5X-1 (GE Healthcare, Ontario, Canada), then overexpressed and purified by standard procedures. For pull- down assays GST-Zasp^Alp^ was added to glutathione paramagnetic beads (Promega, WI, USA) in 20 mM Tris-HCl pH 8, 100 mM NaCl, 1 mM MgCl_2_, 1 mM DTT, 0.2% TritonX-100, 10% glycerol, and incubated for 2 h at 4°C. This was followed by a 1 h blocking step of GST-Zasp^Alp^-coupled beads in the above buffer with 5% BSA. Subsequently, rabbit skeletal muscle α-actinin (Cytoskeleton, CO, USA) was added and incubated for another 2 h at 4°C. Final washes were in the above buffer with 500 mM NaCl and 0.5% TritonX-100. Beads were resuspended in SDS sample buffer and analyzed by SDS-PAGE and immunoblotting. 6×His-Zasp66-RB was coupled to Ni-NTA agarose beads (Qiagen, Ontario, Canada) in 20 mM Tris-HCl pH 8, 100 mM NaCl, 1 mM MgCl_2_, 1 mM DTT, 10 mM Imidazole, 0.2% Triton X-100. α-actinin pull-down and washes were carried out using this buffer.

### Histochemistry and Microscopy

We used the following primary antibodies for immunofluorescent stainings of IFMs: rat anti-Zasp52 [9], mouse anti-α-actinin [55], rabbit anti-obscurin Ig14-16 [56], rat anti-kettin MAC155 [57].

Half thoraces were glycerinated (20 mM Na-Phosphate pH 7.2, 2 mM MgCl_2_, 2 mM EGTA, 5 mM DTT, 0.5% Triton X-100, 50% glycerol) overnight at −20°C. IFMs were dissected, washed in relaxing solution (20 mM Na-phosphate pH 7.2, 5 mM MgCl_2_, 5 mM ATP, 5 mM EGTA) with protease inhibitors, and separated into single myofibrils or left as a whole [56]. Primary antibody incubation was carried out overnight, followed by washes in relaxing solution, and 1–3 h incubations of secondary antibodies and Alexa 594-phalloidin (Life Technologies, Ontario, Canada). Pupal IFMs were dissected in relaxing solution, fixed in 4% paraformaldehyde in relaxing solution, and labeled with primary and secondary antibodies. Antibodies against Zasp52, obscurin and kettin were diluted 1∶100 in relaxing solution. Anti-α-actinin antibody was diluted 1∶10. Fluorescently labeled secondary antibodies of the Alexa series (Life Technologies, Ontario, Canada) were used at 1∶400. Samples were mounted in ProLong Gold antifade solution (Life Technologies, Ontario, Canada).

Images were obtained on a LSM 510 Meta confocal microscope using a 63×1.4 NA Plan Apo oil immersion objective (Carl Zeiss, Germany).

### Electron Microscopy

Thoraces were treated with 5 mM MOPS pH 6.8, 150 mM KCl, 5 mM EGTA, 5 mM ATP, 1% Triton X-100 for 2 h at 4°C, followed by overnight incubation in the same buffer without Triton X-100 but 50% glycerol. This was repeated for a second time. Samples were then washed in rigor solution (5 mM MOPS pH 6.8, 40 mM KCl, 5 mM EGTA, 5 mM MgCl_2_, 5 mM NaN_3_) and fixed in 3% glutaraldehyde, 0.2% tannic acid in 20 mM MOPS pH 6.8, 5 mM EGTA, 5 mM MgCl_2_, 5 mM NaN_3_ for 2 h at 4°C. Secondary fixation and embedding were as described before [15], [20], [56]. For preparation of non-glycerinated samples, hemithoraces were dissected in rigor solution and directly transferred into primary fixative. Images were acquired on a Tecnai 12 transmission electron microscope (FEI, Japan).

## Supporting Information

Figure S1 PDZ domains used for the alignment in Figure 5. Swissprot accession number and amino acid range, as well as rank and score of α-actinin binding to the PDZ domain as predicted by http://sbi.postech.ac.kr/pdz/. Higher rank and score indicate higher predicted α-actinin binding. For *Drosophila* PDZ domains binding partners from *Drosophila* were predicted, for human PDZ domains binding partners from humans were predicted.
(PDF)

Figure S2 Multiple sequence alignment of Zasp PDZ domains using Clustal Omega(1.1.0).
(PDF)

Figure S3 Phylogenetic tree of the sequence alignment in Figure S2 generated with ClustalW2.
(PDF)

Figure S4 Zasp66 gene model and alignment of Zasp66 and Zasp67 with Zasp52. (A) *Zasp66* gene model. On top, genomic region of *Zasp66* with genomic coordinates drawn to scale. Arrows indicate alternative start sites. White boxes, untranslated exons or part of exons; grey boxes, translated exons or part of exons; dark grey boxes, location of two conserved domains, PDZ and Zasp-like motif (ZM). Below, three representative alternatively spliced transcripts. Straight lines indicate constitutive splicing, diagonal lines indicate alternative splicing. The predicted size of splice isoforms is indicated in amino acids and kD. The exon targeted by the RNAi hairpin construct used in this study is indicated by an arrow. (B) Alignment with Clustal Omega of the PDZ domains of Zasp52, Zasp67, and Zasp66 (as found in Zasp66-RB/RK/RM/RF), and the Zasp-like motif (ZM) of Zasp52, Zasp67, and Zasp66. Identical amino acids are highlighted in yellow, similar amino acids are highlighted in grey.
(EPS)

Figure S5 Zasp52 and Zasp67 cooperate to assemble myofibrils. (A) Electron micrographs of IFM of wild type, Dmef2>iZasp52ex20, Dmef2>iZasp67, and Dmef2>iZasp52ex20/iZasp67 double mutants. Global views are shown. Sarcomeres of Dmef2>iZasp67 flies lack Z-disc material to a similar degree as observed in Dmef2>iZasp52ex20 flies. The double mutant shows a more severe disruption of sarcomere structure. Thick and thin filaments are misaligned and Z-discs are severely disrupted. Scale bar, 2 µm. (B) RT-PCR of *Zasp67* and *Tubulin* from wild type and *Zasp67* RNAi knockdown adults at 29°C. (C) qPCR of *Zasp67*, *Tubulin*, and *rp49* from wild type and *Zasp67* RNAi knockdown adults at 29°C. Numbers on the y axis refer to averaged ratios of *Zasp67* mRNA to *Tubulin* and *rp49* mRNAs (normalized to 1 for wild type).
(TIF)

Figure S6 α-actinin still localizes to Z-discs in *Zasp52*, *Zasp66*, and *Zasp67* knockdown flies. Adult IFM myofibrils of wild type, Act88F>iZasp66, Act88F>iZasp52ex20, Act88F>iZasp52ex20/iZasp66, Dmef2>iZasp67, Dmef2>iZasp52ex20, and Dmef2>iZasp52ex20/iZasp67 flies stained with phalloidin (red), anti-α-actinin (magenta), and anti-kettin (green) antibody. α-actinin co-localizes with kettin at the Z-discs in all mutants. Scale bar, 5 µm.
(TIF)

Video S1 GFP-Zasp52 time-lapse recording of embryonic myofibril assembly. One z-section of 241 time points separated by 74 sec was assembled into the movie shown.
(M4V)
