# Supplementary material for: Alp/Enigma Family Proteins Cooperate in Z-Disc Formation and Myofibril Assembly
Source: PLoS Genet. 2013 Mar 7;9(3):e1003342. doi: 10.1371/journal.pgen.1003342 (PMC3591300; doi:10.1371/journal.pgen.1003342)
Supplement: Figure S1 — PDZ domains used for the alignment in Figure 5. Swissprot accession number and amino acid range, as well as rank and score of α-actinin binding to the PDZ domain as predicted by http://sbi.postech.ac.kr/pdz/. Higher rank and score indicate higher predicted α-actinin binding. For Drosophila PDZ domains binding partners from Drosophila were predicted, for human PDZ domains binding partners from humans were predicted. (PDF) [file pgen.1003342.s001.pdf]

Figure S1:

ZASP O75112|1-84 rank 39 score 10.28
MSYSVTLTGPGPWGFRLQGGKDFNMPLTISRITPGSKAAQSQLSQGDLVVAIDGVNTDTMTHLEAQNKIKSASYNLSLTL
QKSK

ENH Q96HC4|2-85 rank 19 score 11.17
SNYSVSLVGPAPWGFRLQGGKDFNMPLTISSLKDGGKAAQANVRIGDVVLSIDGINAQGMTHLEAQNKIKGCTGSLNMTL
QRAS

ENIGMA Q9NR12|1-85 rank 27 score 10.63
MDSFKVVLEGPAPWGFRLQGGKDFNVPLSISRLTPGGKAAQAGVAVGDWVLSIDGENAGSLTHIEAQNKIRACGERLSLG
LSRAQ

PDLIM1 O00151|3-85 rank 69 score 10.27
TQQIDLQGPGPWGFRLVGGKDFEQPLAISRVTPGSKAALANLCIGDVITAIDGENTSNMTHLEAQNRIKGCTDNLTLTVA
RSE

PDLIM4 P50479|1-84 rank 69 score 10.27
MPHSVTLRGPSPWGFRLVGGRDFSAPLTISRVHAGSKAALAALCPGDLIQAINGESTELMTHLEAQNRIKGCHDHLTLSV
SRPE

ALP Q53GG5|1-84 rank 56 score 10.43
MPQTVILPGPAPWGFRLSGGIDFNQPLVITRITPGSKAAAANLCPGDVILAIDGFGTESMTHADAQDRIKAAAHQLCLKI
DRGE

PDLIM2 Q96JY6|1-84 rank 77 score 9.87
MALTVDVAGPAPWGFRITGGRDFHTPIMVTKVAERGKAKDADLRPGDIIVAINGESAEGMLHAEAQSKIRQSPSPLRLQL
DRSQ

Myopodin Q9UMS6|6-88 rank 175 score 7.66
FICISMTGGAPWGFRLQGGKEQKQPLQVAKIRNQSKASGSGLCEGDEVVSINGNPCADLTYPEVIKLMESITDSLQMLIK
RPS

CHAP Q9H987|6-88 rank 71 score 11.27
EVLVTLSGGAPWGFRLHGGAEQRKPLQVSKIRRRSQAGRAGLRERDQLLAINGVSCTNLSHASAMSLIDASGNQLVLTVQ
RLA

Zasp52 A1ZA47|8-90 rank 13 score 8.14
QIKLSRFDAQPWGFRLQGGTDFAQPLLVQKVNAGSLSEQAGLQPGDAVVKINDVDVFNLRHKDAQDIVVRSGNNFVITVQ
RGG

Zasp67 Q9VT49|2-83 rank 22 score 9.33
VLDIKMCRFDNVPWGFRLVGGADYDYPLTVVKVTEGSIADEAGLRVEDIIVRINDTAATPLTHDEAHRLIMGSGSVFYFG
VY

Zasp66 Q7KUB5|8-88 rank 37 score 6.91
FAVVLLRDGQATPWGIRLVGGNDLDTPLIITRVQVGSPAHGELLRGDIISKIGEYDARDLSHADAQQLFRGAGNEIRLVV
H

LMO7 Q8WWI1|1042-1128 rank 953 score 5.38
RISINQTPGKSLDFGFTIKWDIPGIFVASVEAGSPAEFSQLQVDDEIIAINNTKFSYNDSKEWEEAMAKAQETGHLVMDV
RRYGKAG
