# Supplementary material for: Alp/Enigma Family Proteins Cooperate in Z-Disc Formation and Myofibril Assembly
Source: PLoS Genet. 2013 Mar 7;9(3):e1003342. doi: 10.1371/journal.pgen.1003342 (PMC3591300; doi:10.1371/journal.pgen.1003342)
Supplement: Figure S2 — Multiple sequence alignment of Zasp PDZ domains using Clustal Omega(1.1.0). (PDF) [file pgen.1003342.s002.pdf]

Figure S2:

```
ZASP            -MSYSVTLTGPGPWGFRLQGGKDFNMPLTISRITPGSKAAQSQLSQGDLVVAIDGVNTDT
ENH             -SNYSVSLVGPAPWGFRLQGGKDFNMPLTISSLKDGGKAAQANVRIGDVVLSIDGINAQG
ENIGMA          MDSFKVVLEGPAPWGFRLQGGKDFNVPLSISRLTPGGKAAQAGVAVGDWVLSIDGENAGS
PDLIM1          --TQQIDLQGPGPWGFRLVGGKDFEQPLAISRVTPGSKAALANLCIGDVITAIDGENTSN
PDLIM4          -MPHSVTLRGPSPWGFRLVGGRDFSAPLTISRVHAGSKAALAALCPGDLIQAINGESTEL
ALP             -MPQTVILPGPAPWGFRLSGGIDFNQPLVITRITPGSKAAAANLCPGDVILAIDGFGTES
PDLIM2          -MALTVDVAGPAPWGFRITGGRDFHTPIMVTKVAERGKAKDADLRPGDIIVAINGESAEG
Myopodin        --FICISMTGGAPWGFRLQGGKEQKQPLQVAKIRNQSKASGSGLCEGDEVVSINGNPCAD
CHAP            --EVLVTLSGGAPWGFRLHGGAEQRKPLQVSKIRRRSQAGRAGLRERDQLLAINGVSCTN
Zasp52          --QIKLSRFDAQPWGFRLQGGTDFAQPLLVQKVNAGSLSEQAGLQPGDAVVKINDVDVFN
Zasp67          VLDIKMCRFDNVPWGFRLVGGADYDYPLTVVKVTEGSIADEAGLRVEDIIVRINDTAATP
Zasp66          FAVVLLRDGQATPWGIRLVGGNDLDTPLIITRVQVGSPAHG-ELLRGDIISKIGEYDARD
                     :      ***:*: ** :   *: :  :   . :    :   * :  *

ZASP            MTHLEAQNKIKSASYNLSLTLQKSK
ENH             MTHLEAQNKIKGCTGSLNMTLQRAS
ENIGMA          LTHIEAQNKIRACGERLSLGLSRAQ
PDLIM1          MTHLEAQNRIKGCTDNLTLTVARSE
PDLIM4          MTHLEAQNRIKGCHDHLTLSVSRPE
ALP             MTHADAQDRIKAAAHQLCLKIDRGE
PDLIM2          MLHAEAQSKIRQSPSPLRLQLDRSQ
Myopodin        LTYPEVIKLMESITDSLQMLIKRPS
CHAP            LSHASAMSLIDASGNQLVLTVQRLA
Zasp52          LRHKDAQDIVVRSGNNFVITVQRGG
Zasp67          LTHDEAHRLIMGSGSVFYFGVY---
Zasp66          LSHADAQQLFRGAGNEIRLVVH---
                : : ..   .      : : :
```
