# Supplementary figures and images for: Alp/Enigma Family Proteins Cooperate in Z-Disc Formation and Myofibril Assembly
Source: PLoS Genet. 2013 Mar 7;9(3):e1003342. doi: 10.1371/journal.pgen.1003342 (PMC3591300; doi:10.1371/journal.pgen.1003342)

**Figure S3:**

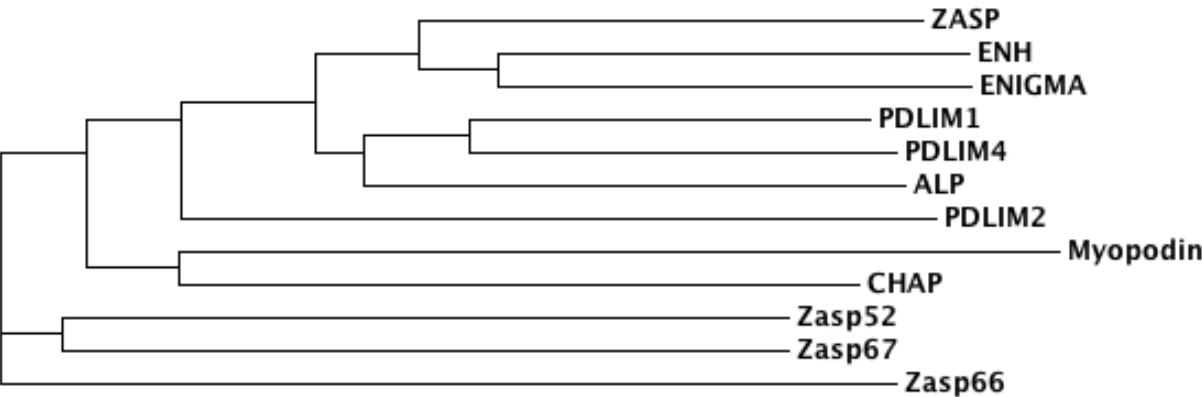

Supplement: Figure S3 — Phylogenetic tree of the sequence alignment in Figure S2 generated with ClustalW2. (PDF) [file pgen.1003342.s003.pdf]

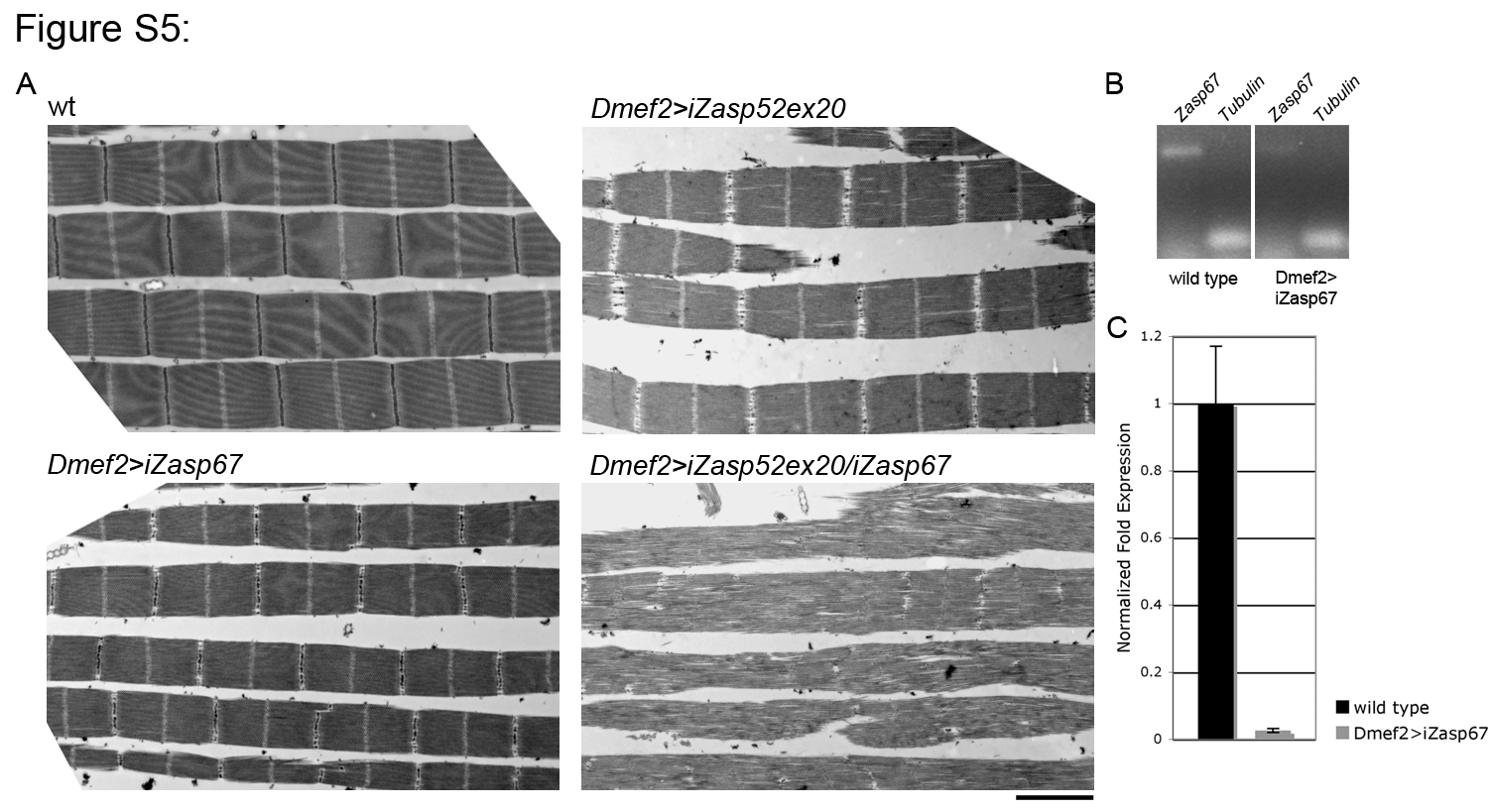

Supplement: Figure S5 — Zasp52 and Zasp67 cooperate to assemble myofibrils. (A) Electron micrographs of IFM of wild type, Dmef2>iZasp52ex20, Dmef2>iZasp67, and Dmef2>iZasp52ex20/iZasp67 double mutants. Global views are shown. Sarcomeres of Dmef2>iZasp67 flies lack Z-disc material to a similar degree as observed in Dmef2>iZasp52ex20 flies. The double mutant shows a more severe disruption of sarcomere structure. Thick and thin filaments are misaligned and Z-discs are severely disrupted. Scale bar, 2 µm. (B) RT-PCR of Zasp67 and Tubulin from wild type and Zasp67 RNAi knockdown adults at 29°C. (C) qPCR of Zasp67, Tubulin, and rp49 from wild type and Zasp67 RNAi knockdown adults at 29°C. Numbers on the y axis refer to averaged ratios of Zasp67 mRNA to Tubulin and rp49 mRNAs (normalized to 1 for wild type). (TIF) [file pgen.1003342.s005.tif]

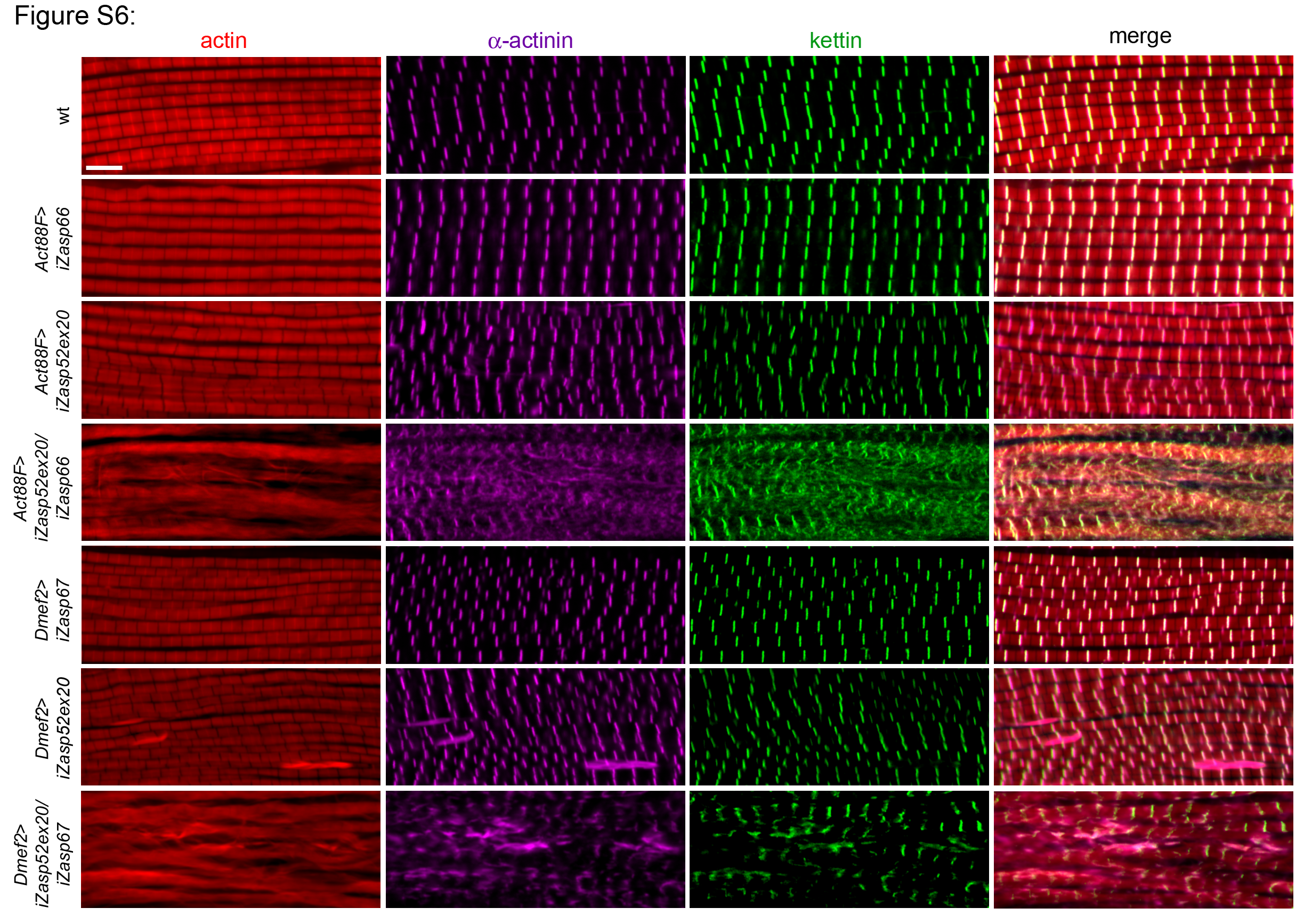

Supplement: Figure S6 — α-actinin still localizes to Z-discs in Zasp52, Zasp66, and Zasp67 knockdown flies. Adult IFM myofibrils of wild type, Act88F>iZasp66, Act88F>iZasp52ex20, Act88F>iZasp52ex20/iZasp66, Dmef2>iZasp67, Dmef2>iZasp52ex20, and Dmef2>iZasp52ex20/iZasp67 flies stained with phalloidin (red), anti-α-actinin (magenta), and anti-kettin (green) antibody. α-actinin co-localizes with kettin at the Z-discs in all mutants. Scale bar, 5 µm. (TIF) [file pgen.1003342.s006.tif]
